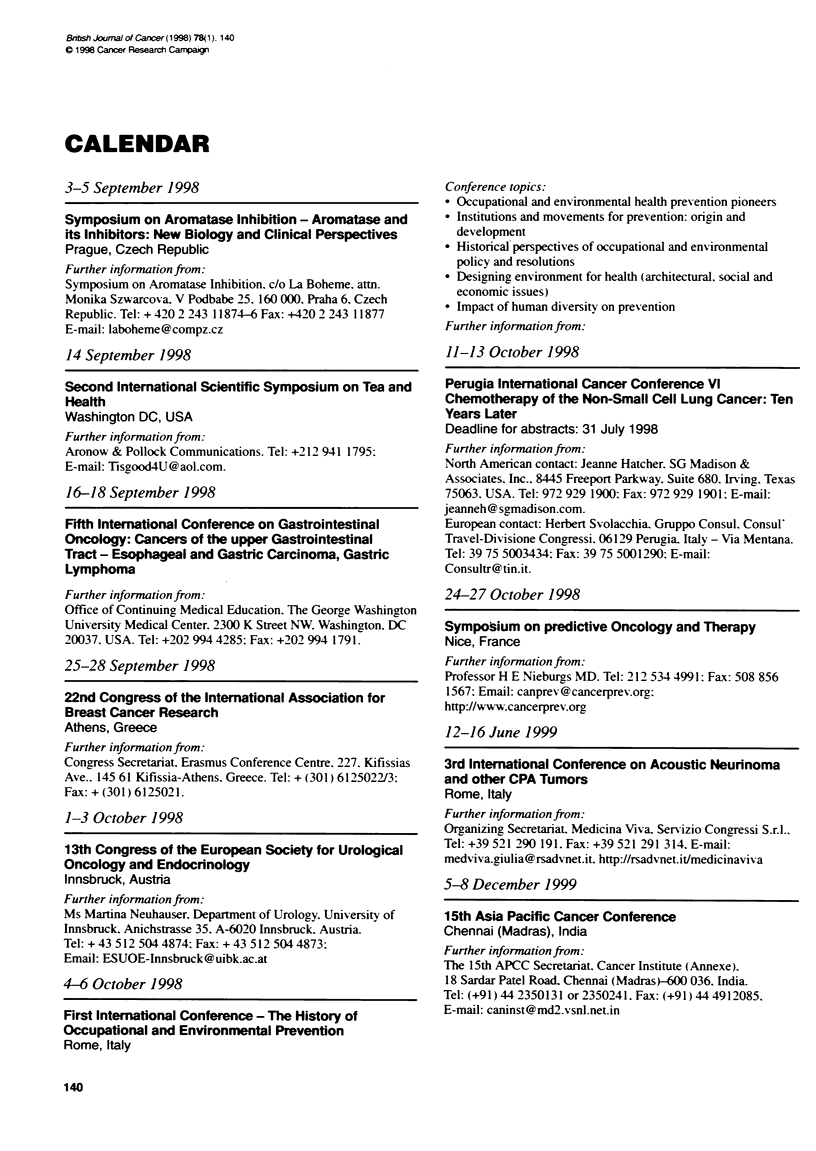# Calendar

**Published:** 1998-07

**Authors:** 


British Journal of Cancer (1998) 78(1). 140

© 1998 Cancer Research Campaign

### CALENDAR

*3–5 September 1998*

**Symposium on Aromatase Inhibition – Aromatase and its Inhibitors: New Biology and Clinical Perspectives**
Prague, Czech Republic

*Further information from:*
Symposium on Aromatase Inhibition. c/o La Boheme. attn. Monika Szwarcova. V Podbabe 25. 160 000. Praha 6. Czech Republic. Tel: + 420 2 243 11874–6 Fax: +420 2 243 11877 E-mail: laboheme@compz.cz

*14 September 1998*

**Second International Scientific Symposium on Tea and Health**
Washington DC, USA

*Further information from:*
Aronow & Pollock Communications. Tel: +212 941 1795: E-mail: Tisgood4U@aol.com.

*16–18 September 1998*

**Fifth International Conference on Gastrointestinal Oncology: Cancers of the upper Gastrointestinal Tract – Esophageal and Gastric Carcinoma, Gastric Lymphoma**

*Further information from:*
Office of Continuing Medical Education. The George Washington University Medical Center. 2300 K Street NW. Washington. DC 20037. USA. Tel: +202 994 4285: Fax: +202 994 1791.

*25–28 September 1998*

**22nd Congress of the International Association for Breast Cancer Research**
Athens, Greece

*Further information from:*
Congress Secretariat. Erasmus Conference Centre. 227. Kifissias Ave.. 145 61 Kifissia-Athens. Greece. Tel: + (301) 6125022/3: Fax: + (301) 6125021.

*1–3 October 1998*

**13th Congress of the European Society for Urological Oncology and Endocrinology**
Innsbruck, Austria

*Further information from:*
Ms Martina Neuhauser. Department of Urology. University of Innsbruck. Anichstrasse 35. A-6020 Innsbruck. Austria.
Tel: + 43 512 504 4874: Fax: + 43 512 504 4873:
Email: ESUOE-Innsbruck@uibk.ac.at

*4–6 October 1998*

**First International Conference – The History of Occupational and Environmental Prevention**
Rome, Italy

*Conference topics:*
- Occupational and environmental health prevention pioneers
- Institutions and movements for prevention: origin and development
- Historical perspectives of occupational and environmental policy and resolutions
- Designing environment for health (architectural. social and economic issues)
- Impact of human diversity on prevention

*Further information from:*

*11–13 October 1998*

**Perugia International Cancer Conference VI Chemotherapy of the Non-Small Cell Lung Cancer: Ten Years Later**
Deadline for abstracts: 31 July 1998

*Further information from:*
North American contact: Jeanne Hatcher. SG Madison & Associates. Inc.. 8445 Freeport Parkway. Suite 680. Irving. Texas 75063. USA. Tel: 972 929 1900: Fax: 972 929 1901: E-mail: jeanneh@sgmadison.com.
European contact: Herbert Svolacchia. Gruppo Consul. Consul' Travel-Divisione Congressi. 06129 Perugia. Italy – Via Mentana. Tel: 39 75 5003434: Fax: 39 75 5001290: E-mail: Consultr@tin.it.

*24–27 October 1998*

**Symposium on predictive Oncology and Therapy**
Nice, France

*Further information from:*
Professor H E Nieburgs MD. Tel: 212 534 4991: Fax: 508 856 1567: Email: canprev@cancerprev.org: http://www.cancerprev.org

*12–16 June 1999*

**3rd International Conference on Acoustic Neurinoma and other CPA Tumors**
Rome, Italy

*Further information from:*
Organizing Secretariat. Medicina Viva. Servizio Congressi S.r.l.. Tel: +39 521 290 191. Fax: +39 521 291 314. E-mail: medviva.giulia@rsadvnet.it. http://rsadvnet.it/medicinaviva

*5–8 December 1999*

**15th Asia Pacific Cancer Conference**
Chennai (Madras), India

*Further information from:*
The 15th APCC Secretariat. Cancer Institute (Annexe). 18 Sardar Patel Road. Chennai (Madras)–600 036. India. Tel: (+91) 44 2350131 or 2350241. Fax: (+91) 44 4912085. E-mail: caninst@md2.vsnl.net.in